# External validation of a prognostic model to improve prediction of psychosis: a retrospective cohort study in primary care

**DOI:** 10.3399/BJGP.2024.0017

**Published:** 2024-10-15

**Authors:** Sarah A Sullivan, Richard Morris, Daphne Kounali, David Kessler, Willie Hamilton, Glyn Lewis, Philippa Lilford, Irwin Nazareth

**Affiliations:** Centre for Academic Mental Health, and National Institute for Health and Care Research Bristol Biomedical Research Centre, University of Bristol, Bristol.; Centre for Academic Primary Care, Population Health Sciences Institute, University of Bristol, Bristol.; Centre for Academic Mental Health, University of Bristol and Oxford Clinical Trials Unit, Botnar Research Centre, University of Oxford, Oxford.; University of Bristol, Bristol.; University of Exeter Medical School, Exeter.; Division of Psychiatry, University College London, London, and National Institute for Health and Care Research Biomedical Research Centre.; Avon and Wiltshire Mental Health Partnership Trust.; Division of Psychiatry, University College London, London.

**Keywords:** early medical intervention, early psychosis, electronic health records, external validation, prediction, primary health care

## Abstract

**Background:**

Early detection could reduce the duration of untreated psychosis. GPs are a vital part of the psychosis care pathway, but find it difficult to detect the early features. An accurate risk prediction tool, P Risk, was developed to detect these.

**Aim:**

To externally validate P Risk.

**Design and setting:**

This retrospective cohort study used a validation dataset of 1 647 934 UK Clinical Practice Research Datalink (CPRD) primary care records linked to secondary care records.

**Method:**

The same predictors (age; sex; ethnicity; social deprivation; consultations for suicidal behaviour, depression/anxiety, and substance misuse; history of consultations for suicidal behaviour; smoking history; substance misuse; prescribed medications for depression/anxiety/post-traumatic stress disorder/obsessive compulsive disorder; and total number of consultations) were used as for the development of P Risk. Predictive risk, sensitivity, specificity, and likelihood ratios were calculated for various risk thresholds. Discrimination (Harrell’s C-index) and calibration were calculated. Results were compared between the development (CPRD GOLD) and validation (CPRD Aurum) datasets.

**Results:**

Psychosis risk increased with values of the P Risk prognostic index. Incidence was highest in younger age groups and, in the main, higher in males. Harrell’s C was 0.79 (95% confidence interval = 0.78 to 0.79) in the validation dataset and 0.77 in the development dataset. A risk threshold of 1.0% gave sensitivity of 65.9% and specificity of 86.6%.

**Conclusion:**

Further testing is required, but P Risk has the potential to be used in primary care to detect future risk of psychosis.

## Introduction

The outcomes of psychosis are often poor.^1^ Several risk factors for a poor outcome — for example, low socioeconomic status^2^ and adverse life events^3^ — are immutable. The duration of untreated psychosis is associated with poorer outcomes,^4^ so the early detection of potentially modifiable risk of psychosis and prevention is necessary.

Most people with psychosis in the UK enter specialist psychosis care via a GP referral. A shorter duration of untreated psychosis is associated with more GP visits prior to diagnosis,^5^ so recognition of early features of psychosis will expedite referral to specialist services. Psychosis diagnoses in GP electronic health records (EHRs) are accurate;^6^ however, GPs under-record more insidious symptoms^7^ — often because they are non-specific.^7^ Furthermore, most GPs see few patients with incident psychosis and are inexperienced in early detection.^8^ There are also referral barriers to specialist psychosis services.^9^ A validated psychosis risk algorithm in primary care could assist timely identification of people at risk. To the authors’ knowledge, there are currently no such risk prediction algorithms to screen the population at scale. Clinical symptom scores and cognitive assessments show promise,^10^ but are costly and too specialised for GPs, and have limited automation potential. An automatable psychosis risk prediction tool using EHRs can benefit patients and alert GPs to early risk identification.

The few studies on psychosis prodrome progression are based on high-risk^11^ patients receiving specialist psychosis treatment. In a previous study,^12^ the authors investigated whether pre-specified symptoms identified attenders who later developed psychosis; they found that specific prodromal behaviours and other psychiatric diagnoses were strongly associated with psychosis. The positive predictive value (PPV) of these factors varied with age and sex. The authors also identified a pattern in consultation frequency for some prodromal behaviours/symptoms up to 5 years before diagnosis, and that people who were later diagnosed with psychosis were more frequent users of GP services than other primary care patients. The authors hypothesised that these candidate predictors could be used to develop an accurate psychosis risk prediction algorithm.

A psychosis risk algorithm, P Risk, was developed.^13^ The discrimination ability was good (Harrell’s C = 0.774) (Supplementary Appendix S7) over 8 years’ follow-up. For a patient with a probability of psychosis risk exceeding 0.5%, sensitivity was 74%, specificity 82%, and the likelihood ratio (LR) was 4.14.^13^ Such an algorithm could be incorporated into primary care EHR systems, allowing automated risk scores to be produced. To estimate clinical utility and transportability, however, risk algorithms must be validated using data not used for their development. This article reports on the external validation of P Risk using a different UK EHR primary care dataset.

**Table:** How this fits in

There is evidence that GPs find it difficult to detect the early warning signs of emerging psychosis. The P Risk algorithm provides primary care with a psychosis risk prediction to help GPs decide whether to refer a patient for a secondary care psychosis assessment or whether to continue to monitor in primary care. This study describes the external validation of the P Risk algorithm. This article reports the external validation of the only psychosis risk prediction algorithm to be used in primary care. External validation of prediction algorithms is essential to provide evidence of transportability — that is, that the algorithm can be used outside of its training environment. This vital step for prediction algorithms is often missed.

## Method

### Design

This retrospective cohort study used GP EHRs reported according to the TRIPOD checklist.^14^

### Data source

Clinical Practice Research Datalink (CPRD) collects longitudinal routinely collected EHRs from UK primary care practices. Practices that use Vision software are incorporated into the CPRD GOLD dataset, which was used to develop P Risk^13^ (the development dataset); other GP surgeries use EMIS software — data from those are incorporated into the CPRD Aurum dataset (the validation dataset), which is used in this study of external validation. The development dataset covered 1 January 2010 until 30 September 2018 and the validation dataset covered 1 January 2010 until 1 July 2020.

### Data linkage

Validation dataset records were individually linked to Hospital Episode Statistics (HES). HES includes UK secondary care records. Validation dataset records were also linked to Office for National Statistics mortality records and Small Area Level statistics (deprivation measures and rural–urban classification).^15^

### Study population and setting

Construction of the external validation cohort was identical to that of the development cohort.^13^ The population of interest included all patients with at least one record of a mental health consultation or mental health prescription within the study period, up to standard (UTS) research quality data,^16^ who were registered with a GP practice in the validation dataset, with at least 5 years’ follow-up data, and links to HES and Index of Multiple Deprivation (IMD) data. In July 2020, there were 35 961 474 research-eligible patients. Of these, 8 884 874 patients had either a mental health symptom or prescription recorded and at least 365 prior days of UTS records.

### Exclusions

**Figure 1.**
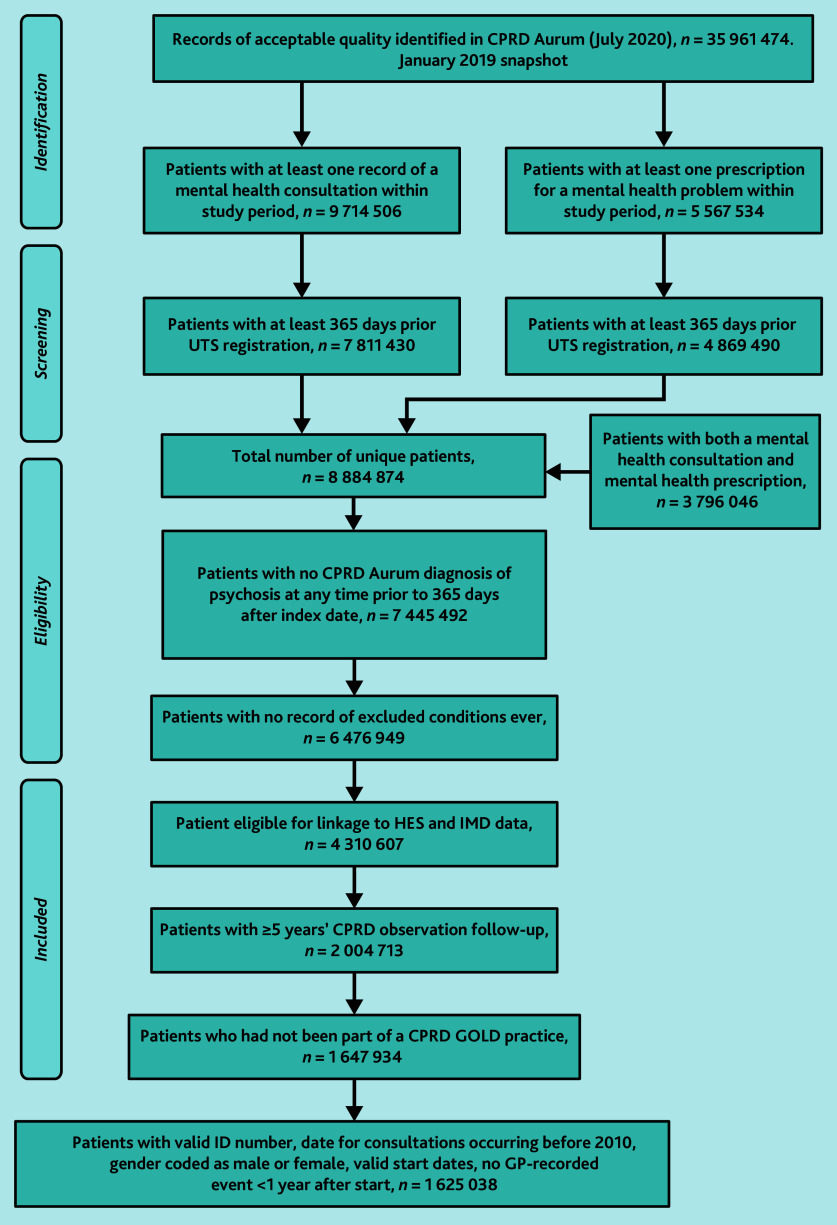
Cohort selection flowchart. CPRD = Clinical Practice Research Datalink. HES = Hospital Episode Statistics. IMD = Index of Multiple Deprivation. UTS = up to standard.

Patients were excluded if they had:

- a record of psychosis (Supplementary Appendix S2) and/or prescription of an antipsychotic (Supplementary Appendix S3) made before, or within, 365 days of the index date, or within 1 year following the index date;
- a record of epilepsy, head injury, dementia, or learning disability (Appendix S4) (Figure 1); and/or
- <5 years of observations within the validation dataset.

This left a sample of 2 004 713 patients. People who were with the development database practices were removed prior to data release, leaving 1 647 934 patient records eligible for linkage from 727 GP practices (Figure 1).

### Inclusions

Patients were included if they had:

- consulted their GP; and/or
- a recorded prescription for a non-psychotic mental health condition (Supplementary Appendix S1).

### Follow-up

Follow-up was from the date of the first consultation after 1 January 2010 until the earliest of:

- recorded date of psychosis diagnosis in primary or secondary care EHRs;
- date of patient death or move from GP practice; or
- 1 July 2020.

### Outcome

The outcome was there being a recorded code for a diagnosis of psychosis according to the International Classification of Diseases, 10th revision (Supplementary Appendix S2):

- in HES;
- in the validation dataset; or
- via a prescription for an appropriate dose of antipsychotics (Supplementary Appendix S3) recorded in the validation dataset.

### Missing data

Predictors that were not recorded were assumed to be missing. The number of outcomes detected was maximised by searching primary and secondary care records, and by using antipsychotic drug prescriptions of doses that were unlikely to have been prescribed for other conditions.

### Model specifications

Using the P Risk model predictors previously developed^13^ (Supplementary Appendix S5), predicted risk over 6 years was calculated for patients whose follow-up time exceeded 6 years or who experienced the outcome within 6 years. Sensitivity, specificity, LRs, and PPVs were calculated for risk thresholds of 0.5%, 1.0%, 1.5%, and 2.0%. Low risk thresholds were chosen as the incidence of psychosis in primary care is low.

### Statistical analysis

Following external validation principles,^17^ Harrell’s C^18^ was calculated to assess discrimination. The authors also assessed prognostic accuracy for prediction of psychosis occurring over 6 years by calculating sensitivity, specificity, PPVs, negative predictive values, and LRs associated with positive and negative predictions. Calibration was assessed by calculating the rate per 100 000 person years overall and within four prognostic groups of the risk score.^13^ Prognostic groups were calculated from whether predicted risk was:

- <mean–1 standard deviation (SD), from the development dataset;
- between mean–1SD and mean;
- between mean and mean+1SD; or
- >mean+1SD.

Age groups were defined according to knot points for cubic splines (an explanation of this term is given in Supplementary Appendix S7) derived from the development dataset. All analyses were carried out in Stata (versions 16 and 17).

## Results

### Validation dataset sample characteristics

The characteristics of the validation dataset sample are given in Supplementary Appendix S6. Of 1 625 038 patients who were followed for a mean of 8.19 years (maximum 10.47 years), 946 without an IMD score and 26 whose event date occurred on, or before, the start date were excluded. There were 4790 (0.3%) psychosis diagnoses during follow-up: 1119 in HES alone, 2736 in the validation dataset alone, and 935 in both. The overall incidence of psychosis was 36.0/100 000 person years. Mean age was 43.6 years (SD = 18.2), median age was 44 years (interquartile range [IQR] = 30–57 years), 52.7% were female, 69.3% were White, and the most frequent index consultation was smoking problems (65.5%), followed by depression and anxiety (27.2%). The proportion of White patients was lower than that reported by the 2021 Office for National Statistics Census 19 Anecdotal evidence suggests that most of the unrecorded ethnicities were likely to be White. This is because GPs are more likely to record ethnicity when it is not the majority ethnicity (that is, White).

### Differences between development and validation datasets

Differences between the development and validation datasets are shown in Supplementary Appendix S6. The average age of the sample in the development dataset was slightly higher and the proportion of females slightly lower than those in the validation dataset. Those in the validation dataset were slightly less likely to be White and had greater levels of socioeconomic deprivation. Patients in the validation dataset were more likely to have an index consultation for smoking problems, suicidal behaviour, and role-functioning problems, and were less likely to have an index consultation for depression and anxiety. There were differences in geographical locations of the patients; these reflected the differing geographical preferences for Vision versus EMIS software among general practices.

### Incidence of psychosis in different follow-up categories

Table 1 shows that risk increases with values of the P Risk prognostic index in both datasets. The incidence of psychosis was fairly similar in both datasets in the lower two prognostic groups (≤16th and 16th–50th centiles) in all follow-up periods, but incidence in the higher two prognostic groups (50th–84th and >84th centile) was greater in the development dataset at all follow-up periods. Table 2 shows that, although the mean score was a little lower in the validation dataset — with a slightly greater proportion of observations in the lowest risk group and slightly fewer in the highest risk group — the distribution of risk scores differed little between the two datasets; this is also shown in Supplementary Appendix S8.

**Table 1.** Incidence of psychosis per 100 000 person years in the development and validation datasets, overall and according to PI (score)

**Prognostic group**	**Psychosis incidence per 100 000 person years, *n***	

	**Development dataset**	**Validation dataset**
**All times**		
Overall	45.3	36.0
PI≤–1.192661 (≤16th centile)	11.2	11.8
–1.192661<PI≤0 (16th–50th centile)	24.8	26.2
0<PI≤1.192661 (50th–84th centile)	56.2	46.8
PI>1.192661 (>84th centile)	159.7	90.0

**0–1.999 years’ follow-up**		
Overall	45.3	36.0
PI≤–1.192661 (≤16th centile)	0	0
–1.192661<PI≤0 (16th–50th centile)	4.8	4.4
0<PI<1.192661 (50th–84th centile)	13.8	10.2
PI>1.192661 (>84th centile)	72.5	32.0

**2–5.999 years’ follow-up**		
Overall	45.3	36.0
PI≤–1.192661 (≤16th centile)	8.3	8.5
–1.192661<PI≤0 (16th–50th centile)	24.2	22.4
0<PI≤1.192661 (50th–84th centile)	81.1	67.6
PI>1.192661 (>84th centile)	322.6	223.0

**≥6 years’ follow-up**		
Overall	45.3	36.0
PI≤–1.192661 (≤16th centile)	18.2	14.8
–1.192661<PI≤0 (16th–50th centile)	43.9	40.5
0<PI≤1.192661 (50th–84th centile)	154.3	105.1
PI>1.192661 (>84th centile)	467.5	309.3

*PI = prognostic index.*

**Table 2.** Distribution of prognostic score in four prognostic groups

**Prognostic group^a^**	**Range**	**Development dataset**		**Validation dataset**	

		**Mean (SD)**	**Observations, *n*^b^ (% of all observations in dataset)**	**Mean (SD)**	**Observations, *n* (% of all observations in dataset)**
Overall	−3.034 to 5.931	0 (1.193)	901 114 (100)	−0.021 (1.201)	5 544 963 (100)
1st	−3.034 to −1.193	−1.731 (0.382)	160 876 (17.9)	−1.734 (0.393)	1 027 691 (18.5)
2nd	−1.193 to 0	−0.535 (0.345)	290 468 (32.2)	−0.559 (0.343)	1 801 223 (32.5)
3rd	0 to 1.193	0.557 (0.339)	305 323 (33.9)	0.566 (0.340)	1 805 944 (32.6)
4th	1.193 to 5.931	1.826 (0.566)	144 447 (16.0)	1.814 (0.542)	910 105 (16.4)

a *Defined according to whether prognostic score fell below mean–1SD (1st prognostic group), between mean–1SD and mean (2nd), between mean and mean+1SD (3rd), or above mean+1SD (4th).*

b *Each participant’s time at risk was split into several intervals, each interval starting at the time when a covariate changed its value (time-updated covariates). SD = standard deviation.*

### Incidence of psychosis according to age, sex, depression, and substance misuse

Data are shown in graphic form in Supplementary Appendix S8. The incidence of psychosis was highest in both datasets in the youngest two age groups, although this was highest for those aged 21.1–39.7 years in the development dataset and for those aged <21.1 years in the validation dataset (Table 3a). In the validation dataset only, a sharp increase in incidence was noted among patients aged >81.1 years compared with younger patients (Table 3a). Incidence in males was higher than in females across both datasets, except for a small increase in females in the validation dataset at >39.7–≤51.0 years (Table 3a).

**Table 3a.** Incidence of psychosis per 100 000 person years, according to age and sex

**Age group^a^**	**Incidence of psychosis per 100 000 person years, *n***			

	**Development dataset**		**Validation dataset**	

	**Males**	**Females**	**Males**	**Females**
≤21.1 years	62.5	60.9	60.9	44.7
>21.1–≤39.7 years	96.1	73.3	50.7	39.1
>39.7–≤51.0 years	56.9	42.0	34.5	35.1
>51.0–≤62.9 years	39.6	28.5	36.7	24.3
>62.9–≤81.1 years	24.7	20.8	32.3	23.3
>81.1 years	29.6	28.8	61.7	46.2

a *Age groupings formed according to knot points in cubic splines.*

The incidence of psychosis according to depression and substance misuse are reported as both were more common in the development dataset but less frequently prescribed for in the validation dataset. In the development dataset, incidence was highest in males with both a reported symptom and prescription for depression; in the validation dataset, it was highest in males with a reported depression symptom only (Table 3b). In both datasets, those with a recorded symptom of substance misuse reported higher incidence of psychosis (Table 3c) than those not reporting this, but the difference was more marked in the development dataset.

**Table 3b.** Incidence of psychosis per 100 000 person years, according to sex and occurrence of depression (by symptoms recorded and/or prescriptions issued)

**Depression**	**Incidence of psychosis per 100 000 person years, *n***			

	**Development dataset**		**Validation dataset**	

	**Males**	**Females**	**Males**	**Females**
None	20.3	19.1	23.0	18.6
Prescription only	100.7	60.2	62.2	31.2
Symptom only	78.3	59.4	113.4	69.5
Prescription and symptom	148.1	73.7	78.4	48.3

**Table 3c.** Incidence of psychosis per 100 000 person years, according to occurrence of substance misuse (by symptom recorded)

**Substance misuse**	**Incidence of psychosis per 100 000 person years, *n***	

	**Development dataset**	**Validation dataset**
No symptom	40.5	32.7
Symptom	138.1	57.2

### Discriminatory power in the validation dataset

Harrell’s C was 0.787 (95% confidence interval [CI] 0.781 to 0.794). Sensitivity and specificity of the risk score were calculated for a series of risk thresholds of predicted probability over 6 years of follow-up. This analysis only included patients who had ≥6 years of follow-up because a shorter follow-up would have been distorted by excluding GP-recorded events occurring before 1 year and a longer follow-up would have omitted a large proportion of the cohort. The same sample selection was used in the authors’ development article.^13^ Using a risk threshold of 1.0% per annum, sensitivity was 65.9% (that is, 1–false negatives), specificity was 86.6% (that is, 1–false positives), LR (positive prediction) was 4.92, and the PPV was 1.04% (that is, ∼1% of test positives against true positives) (Table 4, Supplementary Appendix S8).

**Table 4.** Specificity and sensitivity of prognostic index based on predicted probabilities of developing psychosis over a 6-year period^a^

**Predicted probability of psychosis,%^b^**	**Specificity, %^c^**	**Sensitivity, %^d^**	**Positive LR**	**PPV, %**	**Negative LR**	**NPV, %**
≥0.5	68.4	84.8	2.69	0.57	0.22	99.95
≥1.0	86.6	65.9	4.92	1.04	0.39	99.92
≥1.5	92.6	51.0	6.93	1.46	0.53	99.89
≥2.0	95.4	41.7	9.08	1.91	0.61	99.87

a *3041 events occurred before 6 years; 1 419 883 patients survived 6 years with no psychosis.*

b *Four thresholds not mutually exclusive.*

c *n = 1 419 883.*

d *n = 3041. LR = likelihood ratio. NPV = negative predictive value. PPV = positive predictive value.*

As expected, specificity, positive LR, and PPV increased with the risk threshold, whereas sensitivity decreased and negative LR came closer to 1 (Table 4).

## Discussion

### Summary

Overall incidence of psychosis in the validation dataset was 36.0/100, 000 person years and risk increased with values of the P Risk prognostic index in both datasets. The discrimination accuracy in terms of the Harrell’s C Statistic was slightly better than in the development dataset. This has provided evidence that P Risk detected those at risk of psychosis in primary care with good prognostic performance, and performed well outside of its training dataset.

### Strengths and limitations

This study demonstrates the potential of primary care EHRs to develop useful psychiatric tools. To the authors’ knowledge, there are few databases with primary care records that would allow for the development and validation of a risk algorithm, as has been done here. The authors used a dataset of nearly 2 million records and 4790 diagnoses of psychosis; this is >40 times the recommended number^20^to estimate prognostic accuracy. There are, inevitably, problems with datasets that are not collected for research, but findings can be more easily interpreted if reporting guidelines — such as the TRIPOD checklist^21^ — are followed. Differences between the development and validation datasets were partly due to different clinical records software, which tends to be geographically determined. There were small incidence differences at different age groups in the datasets, which may be because of the different coding systems; however, the authors successfully developed and validated an accurate prediction tool using imperfect data, the arena in which this tool will be used.

To the authors’ knowledge, this is the only published external validation of a primary care psychosis prediction algorithm. Depending on the threshold, the accuracy of P Risk in this validation study is impressive because it predicts with accuracy, without the need for biomarkers or new data. This is valuable in an overstretched primary care system as it uses existing clinical data. In addition, P Risk provides an individual risk prediction whereas, generally, clinical decisions are based on knowledge of average risks. It uses simple predictors that clinicians understand, and its strength is in the complex interaction of these. P Risk is also automatable and can be incorporated into GP systems, such as existing risk algorithms used by GPs for physical health predictors.^22^

P Risk is more accurate at detecting outcomes over >2 years than at outcome occurring at 2 years or before. This is explained by the exclusion of a coded diagnosis of psychosis within a year of cohort entry. Acute-onset psychosis is less common than that with a gradual onset^23^ and may have different predictors; however, the accuracy of P Risk for detecting incident psychosis at 6 years, or even later, presents an opportunity for very early intervention.

It is difficult to be sure of the equivalence of the different coding systems used in the development and validation databases; nevertheless, the discriminatory ability on external validation was impressive. To mitigate against differences in GP recording styles, the authors included codes for prescribed medications to detect predictors and outcomes, which tend to be more accurately recorded. Not all outcomes were confirmed by secondary care data, which may be because the authors only linked to one of three available HES databases; in addition, approximately 20% of patients with psychosis do not have any contact with secondary care services.^24^ The study cohort was older than ideal for detecting new cases of psychosis, but the older age reflects the population who are most likely to seek GP advice. The authors’ eligibility requirement for at least 5 years of follow-up data may have induced a small selection bias by reducing the number of young people, who may be more geographically mobile. Ethnicity was under-recorded by GPs, which could be important because some ethnic groups are at higher risk of psychosis than those of White ethnicity.

### Comparison with existing literature

Overall incidence of psychosis was similar to that in published evidence.^25^ External validation studies in psychiatry are rare, however; a 2017 systematic review of psychosis prediction studies^26^ found published reports of 91 studies, but none had conducted a rigorous validation.

P Risk’s accuracy compares favourably with risk models that use structural neuroimaging methods to detect first-episode psychosis.^27^ As neuroimaging is unlikely to be available to GPs working in primary care, P Risk could prove to be a powerful screening tool to help GPs make referral decisions.

### Implications for research and practice

Psychiatry is undergoing a digital revolution and there is an international move to use EHRs for prediction. It is important that digital tools are rigorously developed and validated to ensure their potential. As P Risk uses data that are already available, it would be low cost and accessible, which demonstrates the importance of exploring the use of digital tools.

This study has shown that P Risk has considerable predictive power in a validation study. The future objective for the tool is that it is automated on GP medical records’ software. The PPV is low, but this is to be expected as PPV depends on the prevalence of psychosis in the population, which is low in a primary care setting. However, the utility of P Risk is as a screening instrument that prompts GPs to consider the possibility of psychosis, which is a fairly uncommon condition in primary care. The opportunity P Risk presents for very early intervention might prompt GPs to hold regular reviews — so-called ‘safety netting’. The only disadvantage of a false positive is that the GP might ask the patient further questions about psychosis and mental health, which is a small price to pay for a timely referral to specialist services, if that is what the GP decides to do.

This study has provided evidence that P Risk is statistically accurate and transferable. It is not yet known whether it could, or would, be used in a live clinical context; the authors are aware of the problem of ‘alert fatigue’,^28^ which might be likely to occur if GPs do not perceive an alert to be useful. Although P Risk is statistically accurate, there is a need for further studies to explore feasibility, acceptability, and impact. The study team is already working on this.

## Data

Clinical Practice Research Datalink (CPRD) Aurum data were supplied by CPRD and Hospital Episode Statistics data were supplied by NHS Digital. All linkages were carried out by the CPRD. All data can be supplied by the CPRD on request. The study datasets cannot be retained by the research group and all datasets have to be deleted after a period of time, as requested by the CPRD.
